# Differences Between Online Trial Participants Who Have Used Statutory Mental Health Services and Those Who Have Not: Analysis of Baseline Data From 2 Pragmatic Trials of a Digital Health Intervention

**DOI:** 10.2196/44687

**Published:** 2023-06-27

**Authors:** Stefan Rennick-Egglestone, Chris Newby, Clare Robinson, Caroline Yeo, Fiona Ng, Rachel A Elliott, Yasmin Ali, Joy Llewellyn-Beardsley, Scott Pomberth, Julian Harrison, Sean P Gavan, Pim Cuijpers, Stefan Priebe, Charlotte L Hall, Mike Slade

**Affiliations:** 1 School of Health Sciences, Institute of Mental Health, University of Nottingham Nottingham United Kingdom; 2 School of Medicine, Institute of Mental Health, University of Nottingham Nottingham United Kingdom; 3 Centre for Evaluation and Methods, Pragmatic Clinical Trials Unit, Queen Mary University of London London United Kingdom; 4 Division of Population Health, Health Services Research & Primary Care, University of Manchester Manchester United Kingdom; 5 Narrative Experiences Online study Lived Experience Advisory Panel Nottingham United Kingdom; 6 Department of Clinical, Neuro and Developmental Psychology, Amsterdam Public Health Research Institute, Vrije Universiteit Amsterdam Amsterdam Netherlands; 7 Unit for Social and Community Psychiatry (WHO Collaborating Centre for Mental Health Service Development), Queen Mary University of London London United Kingdom; 8 National Institute for Health Research MindTech-MedTech Co-operative, Mental Health & Clinical Neurosciences, School of Medicine, Institute of Mental Health, University of Nottingham, Nottingham, United Kingdom Nottingham United Kingdom

**Keywords:** open recruitment, service use, nonservice use, online intervention, online trial, mobile phone

## Abstract

**Background:**

Digital health interventions (DHIs) are an established element of mental health service provision internationally. Regulators have positioned the best practice standard of evidence as an interventional study with a comparator reflective of standard care, often operationalized as a pragmatic trial. DHIs can extend health provision to those not currently using mental health services. Hence, for external validity, trials might openly recruit a mixture of people who have used mental health services and people who have not. Prior research has demonstrated phenomenological differences in mental health experience between these groups. Some differences between service users and nonservice users might influence the change created by DHIs; hence, research should systematically examine these differences to inform intervention development and evaluation work. This paper analyzes baseline data collected in the NEON (Narrative Experiences Online; ie, for people with experience of psychosis) and NEON-O (NEON for other [eg, nonpsychosis] mental health problems) trials. These were pragmatic trials of a DHI that openly recruited people who had used specialist mental health services and those who had not. All participants were experiencing mental health distress. NEON Trial participants had experienced psychosis in the previous 5 years.

**Objective:**

This study aims to identify differences in baseline sociodemographic and clinical characteristics associated with specialist mental health service use for NEON Trial and NEON-O Trial participants.

**Methods:**

For both trials, hypothesis testing was used to compare baseline sociodemographic and clinical characteristics of participants in the intention-to-treat sample who had used specialist mental health services and those who had not. Bonferroni correction was applied to significance thresholds to account for multiple testing.

**Results:**

Significant differences in characteristics were identified in both trials. Compared with nonservice users (124/739, 16.8%), NEON Trial specialist service users (609/739, 82.4%) were more likely to be female (*P*<.001), older (*P*<.001), and White British (*P*<.001), with lower quality of life (*P*<.001) and lower health status (*P*=.002). There were differences in geographical distribution (*P*<.001), employment (*P*<.001; more unemployment), current mental health problems (*P*<.001; more psychosis and personality disorders), and recovery status (*P*<.001; more recovered). Current service users were more likely to be experiencing psychosis than prior service users. Compared with nonservice users (399/1023, 39%), NEON-O Trial specialist service users (614/1023, 60.02%) had differences in employment (*P*<.001; more unemployment) and current mental health problems (*P*<.001; more personality disorders), with lower quality of life (*P*<.001), more distress (*P*<.001), less hope (*P*<.001), less empowerment (*P*<.001), less meaning in life (*P*<.001), and lower health status (*P*<.001).

**Conclusions:**

Mental health service use history was associated with numerous differences in baseline characteristics. Investigators should account for service use in work to develop and evaluate interventions for populations with mixed service use histories.

**International Registered Report Identifier (IRRID):**

RR2-10.1186/s13063-020-04428-6

## Introduction

### Background

Digital health interventions (DHIs) are apps and other forms of interactive technology intended to provide benefits for people experiencing health problems [1]. DHIs for a range of mental health problems have become an established part of mental health service provision internationally. An international survey of health care providers reported that approximately half of the respondents had either discussed or recommended smartphone apps intended to provide benefits for people experiencing bipolar disorder [2]. A 2017 analysis of health service records and systematic web searches identified 13 web applications and 35 smartphone apps available for depression, anxiety, or stress through the National Health Service (NHS) in England [3]. Some mental health DHIs have been used to augment the capabilities of existing services, including a suicide prevention app used by tertiary mental health services in Australia [4] and an app for the management of posttraumatic stress disorder used by a primary care service for US military veterans [5]. DHIs may be delivered with varying degrees of human support, ranging from purely self-directed with no human contact to a technology being used as a means of communication, such as a psychotherapy session delivered through videoconferencing. Meanwhile, there is an abundance of mental health DHIs available directly to the public, frequently in the form of smartphone apps distributed through commercial app stores or subscriptions to self-help websites. Most offerings have no scientific support [6]. Some can recommend harmful strategies, such as the consumption of strong alcohol during a bipolar episode [7].

Health service strategies for making decisions about the commissioning and recommendation of DHIs are evolving. In England, the NHS evaluates DHIs using the Digital Technology Assessment Criteria, which consider clinical safety, data protection, technical assurance, interoperability, usability, and accessibility but not effectiveness. This means that apps can be recommended through the NHS website that have been evaluated as safe but where evidence for effectiveness is not available [8,9]. In parallel, the National Institute for Health and Care Excellence in England has developed an evidence standards framework establishing the minimum and ideal levels of evidence required for recommendation of DHIs [1,10]. This framework considers both effectiveness and cost-effectiveness. An analogous but broader document is the US Food and Drug Administration’s guide on the clinical evaluation of software as a medical device [11]. Within both frameworks, the best practice standard of evidence for DHIs is an interventional study with a comparator reflective of standard care in a current care pathway [1]. This is often operationalized as a randomized controlled trial (RCT) in which a control group receives treatment as usual.

Health care staff have been directly involved in the delivery of treatment in some evaluations of purely self-directed DHIs. In an early RCT of the web application Beating the Blues [12] for anxiety and depression in general practice, a nurse facilitated access to a computer workstation and booked follow-up sessions [13]. In real-world use, support from health care professionals may be unavailable, and hence the external validity of evaluations might be maximized if they are conducted entirely on the web without the involvement of health care professionals [14]. Guidance from UK and US regulators confirms that online recruitment and consent processes are legitimate for all trial types and that consent for a non–clinical trial of an investigational medicinal product can be collected through online forms requiring no identity verification [15,16]. As such, RCTs of self-directed DHIs can be conducted without any planned contact between participants and researchers or health care professionals. We refer to *online trials* where both trial procedures and the intervention are delivered purely online.

Online trials have the potential to increase access to treatment for people with experience of severe mental illness (SMI). SMI is defined as psychological problems that severely affect the ability to engage in functional and occupational activities. This term is collectively used to refer to diagnoses that include schizophrenia, bipolar disorder, and other psychoses [17]. Some people with SMI have never been in contact with specialist mental health services, and a range of large studies suggest that this category might include a substantial majority of the affected population. Taking psychosis as an example, an analysis of community survey data from 18 countries found that mean lifetime prevalence of ever having a psychotic episode was 5.8% (SE 0.2%) [18], and a systematic review found that median lifetime prevalence of ever having a psychotic experience was 7.2% [19]. Both figures are substantially higher than lifetime rates of psychosis service use, which have been estimated as ranging from 0.2% (narrowly defined) to 0.7% (broadly defined) in an epidemiological study on a US community sample [20] and as 1% in a UK NHS policy document [21].

This gap between prevalence and specialist service use may occur for several reasons. First, a range of barriers to accessing traditional mental health services have been identified. A systematic review investigating access for women with perinatal mental illness identified barriers at 4 levels: individual (eg, stigma and poor awareness), organizational (eg, resource inadequacies and service fragmentation), sociocultural (eg, language and cultural barriers), and structural (eg, unclear policy) [22]. Second, people may actively choose not to use specialist services. An interview study has described service avoidance owing to experiences of institutional injustice [23] and prior harmful interactions with health care professionals [24]. Third, people may not perceive mental health problems as illnesses requiring specialist treatment. An interview study included some participants who conceptualized psychosis experiences as having positive qualities [24]. The Hearing Voices Network has challenged current diagnostic systems and positioned supportive communities as an alternative to psychiatric engagement in some cases [25]. Although mental health DHIs may not be of interest to people conceptualizing psychosis experiences as positive, they may have a role in reducing barriers to health service use (eg, by providing a scalable alternative to face-to-face provision in situations where resources are limited or by being approachable for people who have had previous harmful experiences of health services). They may also be accessible to people who challenge the diagnostic system because in many cases there is no inherent need to embed diagnostic concepts into the design of a mental health DHI. Hence, DHIs might extend health service provisions to those who have not previously used statutory mental health services, and the external validity of evaluations might be maximized if investigators plan to openly recruit a mixture of people who have used current mental health services and those who have not.

### Narrative Experiences Online Study

The Narrative Experiences Online (NEON) study [26] has investigated whether receiving online access to recorded mental health recovery narratives improves quality of life in people affected by mental health problems. The study has developed a DHI called the NEON Intervention, a web application delivering automated access to the NEON Collection of recorded mental health recovery narratives. The design of the NEON Intervention has been informed by the NEON Impact Model, a primarily transdiagnostic change model [27] developed from a theory base on the impact of recovery narratives on recipients [28-31]. NEON has conducted 3 pragmatic RCTs to evaluate the NEON Intervention [32]. The NEON Trial (ISRCTN11152837) is a definitive RCT for people with experience of psychosis. The second trial is a definitive RCT for people with experience of other (eg, nonpsychosis) mental health problems (NEON for other [eg, nonpsychosis] mental health problems [NEON-O] Trial; ISRCTN63197153). A third RCT evaluating the feasibility of using the NEON Intervention with people with experience of caring for people with mental health problems (NEON for people with experience of caring for people with mental health problems [NEON-C] Trial; ISRCTN76355273) will be reported elsewhere. The NEON Intervention is fully automated and could be delivered on a large scale without diagnosis by health care professionals. Hence, to maximize external validity, inclusion criteria and recruitment strategies for the NEON Trial and NEON-O Trial enabled the *open recruitment* [33] of people with current or prior experience of mental health services and those without.

A specific consideration for mental health RCTs with open recruitment is whether participants with different histories of mental health service use have different characteristics at baseline. Some differences between service users and nonservice users can influence the change created by DHIs; hence, research should systematically examine these differences to inform the development and evaluation of DHIs. For psychosis, existing evidence raises the possibility of differences in symptomatology between people who have used specialist mental health services and those who have not. A study comparing current service users diagnosed with a psychotic disorder with people reporting persistent psychosis experiences and no history of service use found that the people in the latter group were less paranoid, with fewer cognitive difficulties and more intense somatic or tactile hallucinations [34]. A study comparing the phenomenology of hallucinations reported by current service users diagnosed with schizophrenia and those reported by nonservice users experiencing hallucinations found a substantial difference in the character of hallucinations described [35]. Survey evidence demonstrates that psychoeducation is routinely provided to mental health service users [36,37], and hence service users may develop differences in knowledge and attitudes about mental health through experiencing psychoeducation. This is relevant, given that existing attitudes can influence DHI experiences [38] and predicted the outcome in 1 online trial [39].

### Aims of the Study

The NEON Trial and the NEON-O Trial have openly recruited large samples. This study aims to identify differences in baseline sociodemographic and clinical characteristics that are associated with participants’ history of statutory mental health service use. The objectives were to (1) report the baseline characteristics of participants in both trials, (2) compare the baseline characteristics of participants in each trial who have used statutory specialist mental health services with those of the participants who have not, and (3) compare the baseline characteristics of NEON Trial participants currently using specialist services with those of prior users.

## Methods

### Ethics Approval

Ethics approval was obtained in advance from the Leicester Central Research Ethics Committee (19/EM/0326).

### Mental Health Lived Experience Involvement

Authors with lived experience of SMI, including the lead author, were involved in the conceptualization and presentation of the work. An involvement plan was coproduced to enable the meaningful involvement of authors with experience of mental illness who were not employed as researchers (Multimedia Appendix 1).

### Participants

The inclusion criteria shared by the NEON Trial and NEON-O Trial were as follows: experience of mental health–related distress in previous 6 months, resident in England, aged ≥18 years, capable of accessing or being supported to access the internet (on a PC, a mobile device, or at a community venue), able to understand written and spoken English, and capable of providing online informed consent. In addition, the NEON Trial only included people with experience of psychosis in the last 5 years, and the NEON-O Trial only included participants with experience of any other mental health problems in the last 5 years.

### Baseline Measures and Sociodemographic Items

Six measures were collected at baseline in both trials. Eleven sociodemographic items were collected in both trials. An additional service use item was collected in the NEON Trial. All data were collected through online forms with automated validation of responses. These forms are described in Multimedia Appendix 2. The design and implementation of the online forms were initially validated through a feasibility study with current users of statutory mental health services (n=25) [27] and subsequently validated through the NEON Trial internal pilot [32].

The outcomes assessed through the collected measures were quality of life, psychological distress, hope, self-efficacy, meaning in life (presence and search), and health status. Quality of life was assessed using the Manchester Short Assessment (MANSA), which comprises 12 subjective items self-rated on a Likert scale ranging from 1 to 7. The MANSA score is the mean, ranging from 1 (low quality of life) to 7 (high quality of life). The MANSA has adequate psychometric properties [40] and is widely used as a primary outcome in psychosis research [41]. Psychological distress was assessed using the Clinical Outcomes in Routine Evaluation-10 (CORE-10), a 10-item measure developed from the 34-item Clinical Outcomes in Routine Evaluation–Outcome Measure (CORE-OM), with sum scores ranging from 0 (low distress) to 40 (high distress). The CORE-10 has high internal consistency (Cronbach α=.90) and high correlation with the CORE-OM in a clinical sample (*r*=0.94) [42]. Hope was assessed using the Herth Hope Index (HHI), a 12-item measure developed from the 30-item Herth Hope Scale, with sum scores ranging from 12 (low hope) to 48 (high hope). The HHI has high internal consistency (Cronbach α=.97), high test-retest reliability (Cronbach α=.91), and high correlation with the Herth Hope Scale in a clinical sample (*r*=0.92) [43]. Self-efficacy was assessed through the Mental Health Confidence Scale (MHCS), a 16-item measure with sum scores ranging from 16 (low self-efficacy) to 96 (high self-efficacy). The MHCS has high internal consistency (Cronbach α=.94) [44]. Meaning in life was assessed through the Meaning in Life Questionnaire, a 10-item measure of the presence and search for life producing 2 mean scores, *presence* and *search*, ranging from 1 (low) to 7 (high). Both subscales had good internal consistency in a student population (presence: Cronbach α=.86 and search: Cronbach α=.87) [45]. Health status was assessed through the 5-item EQ-5D-5L [46]. Following National Institute for Health and Care Excellence guidelines on health technology evaluations [47], EQ-5D-5L data were mapped to EQ-5D-3L health utility values using an established mapping function parameterized on age and sex [48] and a data set collected through a population study in the UK [49]. Higher values indicate better health.

The demographic items were as follows: age (in years), gender (female, male, and other), ethnicity, region of residence, highest educational qualification, lifetime use of primary care mental health services, lifetime use of specialist mental health services, current use of mental health services in relation to psychosis (NEON Trial only), main mental health problem in last month, best description of recovery status, residential status, and employment status. Response options for ethnicity and highest educational qualification followed UK Office for National Statistics guidance for England [50,51]. Assessment of residential and employment statuses was performed through 2 questions modified from section 2 of the MANSA. Mental health service use was assessed through 3 questions developed by the study team, which were numbered as on our demographics form (the text in the questions accurately reports the demographics form, but the Improving Access to Psychological Therapies programme was misnamed in Q6):

Q6: Have you ever (including currently) used primary care mental health services, e.g. had support or medication prescribed by your GP [general practitioner] for mental health issues, seen a GP practice counsellor, or used the Increasing Access to Psychological Therapies (IAPT) programme?

yes, no

Q7: Have you ever (including currently) used specialist mental healthcare services, e.g. a community mental health team, mental health in-patient ward?

yes, no

Q8: [NEON Trial only] Which of the following best describes the current contact you have with the NHS about your experiences of psychosis? (No contact with any NHS service, Contact with my GP, Contact with primary care counsellor, Contact with Improving Access to Psychological Therapies (IAPT), Contact with a specialist community mental health team, Currently a mental health in-patient in hospital)

one only

### Procedures

Participants were recruited from across England from March 9, 2020 (both trials), to March 1, 2021 (NEON Trial), and March 26, 2021 (NEON-O Trial). Although most of the participants were recruited online, the trials used a mixed online and offline approach to participant recruitment. Recruitment was performed through paid online advertising on mental health websites (GBP £1900 [US $2360] spend) and printed magazines (GBP £2200 [US $2730] spend); promotional messaging distributed by mental health community groups (n=775) and primary health care practices (n=66); promotional messaging distributed on Facebook, Twitter, and Google (GBP £10,300 [US $ 12,790] spend to enhance the reach of messages); media appearances by the central study team; and the work of clinical research officers at 11 secondary care mental health trusts. Community groups were identified by the central study team, including through personal knowledge and searches of publicly available information. They were contacted by a study team administrator and offered approved promotional messaging to distribute. Clinical research officers approached potential participants in person and distributed promotional messaging through local authorized channels such as mailing lists of service users who had consented to be contacted about research studies. All promotional advertising and messaging conformed to principles approved in advance by the supervising research ethics committee [52]. Recruitment was reviewed by a program steering committee 4 months after the trials opened. The program steering committee recommended specific effort to increase recruitment of people from minoritized groups. This was enacted through targeted recruitment messaging and the employment of paid community champions. Both trials were powered on the MANSA score, which was the primary outcome. The target samples were 684 for the NEON Trial [32] and 994 for the NEON-O Trial [53].

After recruitment, there was no planned human contact. All recruitment approaches directed potential participants to an online eligibility-checking interface that requested responses to a series of questions specified in the trial protocol [32]. There was no planned supervision from researchers or health care practitioners; hence, all responses were self-rated. No formal diagnosis of a mental health condition was required for participation. Participants in the NEON Trial responded *yes* to a question selected to be accessible to people who had not received a diagnosis: “In the last 5 years have you had experiences diagnosed as psychosis, or that you or others would call psychosis (such as seeing or hearing things that others have not, or having unusual beliefs that other people disagree with)?” [24]. Experience of mental health distress in the last 6 months was evaluated using 3 items from the Threshold Assessment Grid [54].

Eligible potential participants were provided with access to an online participant information sheet that included contact details for the study team in case clarification was needed. Participants completed an online informed consent form by providing an email address and optional telephone number. The consent process was concluded by clicking a link in an auto-generated email to validate the email address. After confirming consent, participants completed online forms to enable collection of baseline demographic information and completed all measures (MANSA, CORE-10, HHI, MHCS, Meaning in Life Questionnaire, and EQ-5D-5L). After form completion, participants were randomized using a web-based system validated by a clinical trials unit to intervention or control arms. All data considered in this paper are prerandomization data.

### Statistical Analysis Plan

The significance threshold was α=.05 throughout. Before planning the analysis, a chi-square test was conducted to compare baseline main mental health problem between the 2 trials. There was a significant difference (*P*<.001); hence, the 2 trials were analyzed separately.

For objective 1, demographic and clinical characteristics of participants randomized to each trial were described via means and SDs or medians and IQRs for normally distributed and nonparametric continuous data, respectively. Categorical data were described through counts with percentages. Descriptive statistics were calculated for all baseline demographic items and for the index scores for all measures. Ethnicity responses were grouped into 2 categories (White British and Other ethnicity) because of the small numbers of participants in most ethnicity categories and because minority identities historically predict mental health problems, mediated by minority stress [55]. To produce EQ-5D-3L values, a published library was used to map participant responses [48]. The mapping algorithm used a binary model for participant sex (female and male), whereas demographic data collected in the NEON trials were for *gender*, using a nonbinary model (female, male, and other). As sex and gender are separate constructs [56], we treated EQ-5D values as missing for participants responding *other* for gender, while acknowledging that this limits the generalizability of the EQ-5D findings and excludes some of the participants. The mapping algorithm also required age; hence, EQ-5D values were treated as missing where age was not available.

For objective 2, demographic information on service use was used to partition trial participants into 1 of 2 disjoint categories. *Specialist service use* participants answered yes to Q7, with all other trial participants (including those who had not used specialist services or only used primary care mental health services) categorized as *no specialist service use*. Hypothesis testing for differences on the remaining demographic items and index scores was conducted, excluding participants with missing service use data (<1%). We used 2-tailed *t* tests and Mann-Whitney tests for normally distributed data and nonparametric continuous data, respectively. The chi-square or Fisher exact test, where appropriate, was used for categorical data. To adjust for multiple testing of 18 variables for the NEON Trial and 17 variables for the NEON-O Trial, the significance thresholds were adjusted to α=.0028 and α=.0029, respectively (Bonferroni correction). Participants with a gender identity other than female or male were excluded for hypothesis testing owing to the small numbers.

For objective 3, responses to Q8 were used to partition NEON Trial participants in the *specialist service use* group into 2 disjoint subcategories: *current use* (participant is currently using specialist mental health services) and *prior use* (participant has previously used such services but is not currently using them). This information was not collected for NEON-O Trial participants. Testing for significant differences was as for objective 2, but the Bonferroni correction was for 16 variables. This established a significance threshold of α=.0031.

Following UK Data Service guidance on statistical disclosure [57], rows with cells containing between 1 and 4 participants were merged into other rows for presentation of results only (hypothesis testing was unaffected). This necessitated the merging of at least 1 other row so that the true value could not be inferred. The exceptions were *gender* and *best description of recovery*, where rows were not combined.

## Results

### Objective 1 (Baseline Characteristics)

Both trials overrecruited in relation to their preplanned target samples. For the NEON Trial, 739 participants were recruited. Baseline sociodemographic and clinical characteristics of NEON Trial participants are shown in Table 1.

Baseline measures are shown in Table 2.

For the NEON-O Trial, 1023 participants were recruited. Baseline sociodemographic and clinical characteristics of NEON-O Trial participants are presented in Table 3.

Baseline measures for NEON-O Trial participants are presented in Table 4.

**Table 1:** Baseline sociodemographic and clinical characteristics of Narrative Experiences Online Trial participants.

		Baseline (N=739)	Specialist service use comparison (for those with service use data)			
			Specialist service use (n=609)	No specialist service use (n=124)	*P* value	
**Gender, n (%)**						*<.001* ^a^
	Female	443 (59.9)	392 (64.4)	51 (41.1)		
	Male	274 (37.1)	206 (33.8)	68 (54.8)		
	Other	16 (2.2)	11 (1.8)	5 (4)		
Age (years), mean (SD)		34.8 (12.0)	35.9 (12.4)	29.4 (8.2)	*<.001*	
**Ethnicity, n (%)**						*<.001*
	White British	561 (75.9)	483 (79.3)	78 (62.9)		
	Other ethnicity	172 (23.5)	126 (20.7)	46 (37.1)		
**Region of residence, n (%)**						*<.001*
	East of England	53 (7.2)	43 (7.1)	10 (8.1)		
	London	166 (22.5)	120 (19.7)	46 (37.1)		
	Midlands	112 (15.2)	92 (15.1)	20 (16.1)		
	North East and Yorkshire	80 (10.8)	69 (11.3)	11 (8.9)		
	North West	66 (8.9)	54 (8.9)	12 (9.7)		
	South East	133 (18)	119 (19.5)	14 (11.3)		
	South West	123 (16.6)	112 (18.4)	11 (8.9)		
**Highest educational qualification, n (%)**						.18
	No qualification	51 (6.9)	42 (6.9)	9 (7.3)		
	O levels or GCSEs^b^	117 (15.8)	100 (16.4)	17 (13.7)		
	A levels, AS^c^ levels, NVQs^d^, or equivalent	278 (37.6)	222 (36.5)	56 (45.2)		
	Degree-level qualification	207 (28)	172 (28.2)	35 (28.2)		
	Higher degree–level qualification	80 (10.8)	73 (12)	7 (5.6)		
**Living status, n (%)**						.05
	Alone	215 (29.1)	185 (30.4)	26 (21)		
	With others	524 (70.9)	424 (69.6)	98 (79)		
**Employment status, >n (%)**						*<.001*
	Employed or sheltered employment^e^	287 (38.9)	208 (34.2)	76 (61.3)		
	Training and education	76 (10.3)	59 (9.7)	17 (13.7)		
	Unemployed or retired^e^	376 (50.9)	342 (56.2)	31 (25)		
**Current mental health problem, n (%)**						*<.001*
	Don’t want to say or did not experience mental health problems^e^	39 (5.3)	31 (5.1)	8 (6.4)		
	Developmental disorder or eating disorder^e^	30 (4)	22 (3.6)	8 (6.4)		
	Mood disorder	265 (35.9)	215 (35.3)	50 (40.3)		
	Personality disorder	138 (18.7)	123 (20.2)	15 (12.1)		
	Schizophrenia or other psychosis	154 (20.8)	147 (24.1)	7 (5.6)		
	Stress-related disorders	82 (11.1)	59 (9.7)	23 (18.5)		
	Substance-related disorder	25 (3.4)	12 (2)	13 (10.5)		
**Lifetime user of primary care mental health services, n (%)**						*<.001*
	Yes	698 (94.5)	595 (97.7)	103 (83.1)		
	No	35 (4.7)	13 (2)	21 (16.9)		
**Current use of mental health services for psychosis, n (%)**						*<.001*
	No contact with any NHS^f^ service	100 (13.5)	74 (12.2)	26 (21)		
	General practitioner	234 (31.7)	186 (30.5)	48 (38.7)		
	Primary care counsellor	59 (8)	42 (6.9)	17 (13.7)		
	IAPT^g^	56 (7.6)	39 (6.4)	17 (13.7)		
	Specialist community mental health team or mental health in-patient in hospital	279 (37.7)	263 (43.2)	16 (12.9)		
**How would you best describe your recovery?, n (%)**						*<.001*
	Don’t want to say	48 (6.5)	36 (5.9)	12 (9.7)		
	Not yet thinking about recovery	91 (12.3)	63 (10.3)	28 (22.6)		
	Working on recovery	510 (69)	428 (70.3)	82 (66.1)		
	Living beyond disability	84 (11.4)	82 (13.5)	2 (1.6)		

^a^Significant *P* values are highlighted in italics (α=.0028).

^b^GCSE: General Certificate of Secondary Education.

^c^AS: advanced subsidiary.

^d^NVQ: National Vocational Qualification.

^e^Rows have been merged to avoid participant identifiability where cell counts are <5.

^f^NHS: National Health Service.

^g^IAPT: Improving Access to Psychological Therapies.

**Table 2:** Baseline measures for Narrative Experiences Online Trial participants.

	Baseline (N=739)	Specialist service use comparison (for those with service use data)		
		Specialist service use (n=609)	No specialist service use (n=124)	*P* value
MANSA^a^, mean (SD)	3.7 (0.9)	3.6 (1)	4 (0.8)	*<.001* ^b^
CORE-10^c^, mean (SD)	22.7 (7.3)	22.7 (7.6)	22.7 (5.6)	.98
HHI^d^, mean (SD)	28.7 (6.8)	28.7 (6.9)	28.4 (6.4)	.69
MHCS^e^, mean (SD)	49.9 (14.2)	49.7 (14.5)	51.1 (12.8)	.28
MLQ^f^ (presence subscale), mean (SD)	3.5 (1.4)	3.4 (1.5)	3.6 (1.1)	.32
MLQ (search subscale), mean (SD)	4.6 (1.4)	4.7 (1.4)	4.3 (1.2)	.005
EQ-5D-3L, median (IQR)	0.5 (0.4)	0.5 (0.4)	0.6 (0.3)	*.002*

^a^MANSA: Manchester Short Assessment.

^b^Significant *P* values are highlighted in italics (α=.0028).

^c^CORE-10: Clinical Outcomes in Routine Evaluation-10.

^d^HHI: Herth Hope Index.

^e^MHCS: Mental Health Confidence Scale.

^f^MLQ: Meaning in Life Questionnaire.

**Table 3:** Baseline sociodemographic and clinical characteristics for Narrative Experiences Online for other (eg, nonpsychosis) mental health problems Trial participants.

			Baseline (N=1023)		Specialist service use comparison (for those with service use data)				
					Specialist service use (n=614)		No specialist service use (n=399)		*P* value
**Gender, n (%)**									.46
	Female	811 (79.3)		494 (80.5)		317 (79.4)			
	Male	184 (18)		106 (17.3)		78 (19.5)			
	Other	18 (1.8)		14 (2.3)		4 (1)			
Age (years), mean (SD)			38.4 (13.6)		38.8 (13.6)		37.7 (13.5)		.19
**Ethnicity, n (%)**									.02
	White British	827 (80.8)		516 (84)		311 (77.9)			
	Other ethnicity	185 (18.1)		97 (15.8)		87 (21.8)			
**Region of residence, n (%)**									.03
	East of England	61 (6)		36 (5.9)		25 (6.3)			
	London	210 (20.5)		108 (17.6)		102 (25.6)			
	Midlands	203 (19.8)		125 (20.4)		78 (19.5)			
	North East and Yorkshire	102 (10)		60 (9.8)		42 (10.5)			
	North West	98 (9.6)		67 (10.9)		31 (7.8)			
	South East	214 (20.9)		144 (23.5)		70 (17.5)			
	South West	125 (12.2)		74 (12.1)		51 (12.8)			
**Highest educational qualification, n (%)**									.51
	No qualification	30 (2.9)		18 (2.9)		12 (3)			
	O levels or GCSEs^a^	116 (11.3)		79 (12.9)		37 (9.3)			
	A levels, AS^b^ levels, NVQs^c^, or equivalent	327 (32)		194 (31.6)		133 (33.3)			
	Degree-level qualification	349 (34.1)		208 (33.9)		141 (35.3)			
	Higher degree–level qualification	191 (18.7)		115 (18.7)		76 (19)			
**Living status, n (%)**									.003
	Alone	229 (22.4)		156 (25.4)		69 (17.3)			
	With others	794 (77.6)		458 (74.6)		330 (82.7)			
**Employment status, n (%)**									*<.001* ^d^
	Employed or sheltered employment^e^	592 (57.9)		326 (53.1)		260 (65.2)			
	Training and education	106 (10.4)		68 (11.1)		38 (9.5)			
	Unemployed	272 (26.6)		184 (30)		85 (21.3)			
	Retired	53 (5.2)		36 (5.9)		16 (4)			
**Current mental health problem, n (%)**									*<.001*
	Don’t want to say	14 (1.4)		9 (1.5)		5 (1.3)			
	Did not experience mental health problems	31 (3)		11 (1.8)		20 (5)			
	Eating disorder	45 (4.4)		37 (6)		8 (2)			
	Mood disorder	626 (61.2)		326 (53.1)		300 (75.2)			
	Personality disorder	123 (12)		113 (18.4)		10 (2.5)			
	Stress-related disorders	152 (14.9)		102 (16.6)		50 (12.5)			
	Developmental disorder, schizophrenia or other psychosis, or substance-related disorder^e^	22 (2.2)		16 (2.6)		6 (1.6)			
**Lifetime user of primary care mental health services, n (%)**									*<.001*
	Yes	949 (92.8)		598 (97.4)		351 (88)			
	No	64 (6.2)		16 (2.6)		48 (12)			
**How would you best describe your recovery?, n (%)**									.25
	I don’t want to say	64 (6.3)		34 (5.5)		30 (7.5)			
	Not yet thinking about recovery	64 (6.3)		35 (5.7)		29 (7.3)			
	Working on recovery	784 (76.6)		488 (79.5)		296 (74.2)			
	Living beyond disability	101 (9.9)		57 (9.3)		44 (11)			

^a^GCSE: General Certificate of Secondary Education.

^b^AS: advanced subsidiary.

^c^NVQ: National Vocational Qualification.

^d^Significant *P* values are highlighted in italics (α=.0029).

^e^Rows have been merged to avoid participant identifiability where cell counts are <5.

**Table 4:** Baseline measures for Narrative Experiences Online for other (eg, nonpsychosis) mental health problems Trial participants.

	Baseline (N=1023)	Specialist service use comparison (for those with service use data)		
		Specialist service use (n=614)	No specialist service use (n=399)	*P* value
MANSA^a^, mean (SD)	3.8 (0.9)	3.7 (0.9)	4 (0.9)	*<.001* ^b^
CORE-10^c^, mean (SD)	21.6 (7.3)	22.6 (7.2)	20.1 (7.1)	*<.001*
HHI^d^, mean (SD)	28.9 (6.9)	28 (6.8)	30.3 (6.8)	*<.001*
MHCS^e^, mean (SD)	51.6 (14.2)	49.1 (13.9)	55.4 (13.7)	*<.001*
MLQ^f^ (presence subscale), mean (SD)	3.4 (1.5)	3.3 (1.5)	3.7 (1.5)	*<.001*
MLQ (search subscale), mean (SD)	4.7 (1.4)	4.7 (1.5)	4.6 (1.4)	.79
EQ-5D-3L, median (IQR)	0.6 (0.4)	0.6 (0.4)	0.7 (0.3)	*<.001*

^a^MANSA: Manchester Short Assessment.

^b^Significant *P* values are highlighted in italics (α=.0029).

^c^CORE-10: Clinical Outcomes in Routine Evaluation-10.

^d^HHI: Herth Hope Index.

^e^MHCS: Mental Health Confidence Scale.

^f^MLQ: Meaning in Life Questionnaire.

### Objective 2 (Specialist Service Use vs No Specialist Service Use)

NEON Trial participants were partitioned into *specialist service use* (609/739, 82.4%) and *no specialist service use* (124/739, 16.8%), with missing service use data for 0.8% (6/739) of the participants. Comparisons of these 2 NEON Trial subgroups are shown in Tables 1 and 2 (columns 3-5). After Bonferroni correction to a significance threshold of α=.0028, a range of comparisons were identified as significant. NEON Trial participants who were current or previous users of specialist services, compared with those who were not, were more likely to be female (*P*<.001), older (*P*<.001), and White British (*P*<.001). They had lower quality of life (*P*<.001) and lower health status (*P*=.002). There were differences in current mental health problems (*P*<.001), with a higher percentage of specialist service users experiencing psychosis or personality disorders and a lower percentage of specialist service users experiencing mood disorders, stress disorders, or substance disorders. There were differences in employment status (*P*<.001; including a higher percentage of unemployment among specialist service users). There were also differences in geographical distribution (*P*<.001), with a higher percentage of specialist service users residing in South West England and South East England (excluding London) and a lower percentage of specialist service users residing in London. There were differences in recovery status (*P*<.001), with specialist service users self-identifying as being further through the recovery process. Specialist service users were more likely to have accessed primary care mental health services (*P*<.001), but this is close to being tautologous because, in the UK system, most referrals into specialist mental health services are through primary care.

NEON-O Trial participants were partitioned into *specialist service use* (614/1023, 60.02%) and *no specialist service use* (399/1023, 39%), with missing service use data for 0.98% (10/1023) of the participants. Comparisons of these 2 subgroups are shown in Tables 3 and 4 (columns 3-5). After Bonferroni correction to a significance threshold of α=.0029, a range of comparisons were identified as significant. NEON-O Trial participants who were current or previous users of specialist services, compared with those who were not, had lower quality of life (*P*<.001), were more distressed (*P*<.001), were less hopeful (*P*<.001), were less empowered (*P*<.001), had less meaning in life (*P*<.001), and had lower health status (*P*<.001). They had differences in current mental health problems (*P*<.001), with a higher percentage of specialist service users experiencing personality disorders and a lower percentage of specialist service users experiencing mood disorders. They had differences in employment status (*P*<.001), with a higher percentage of specialist service users being unemployed. They were more likely to have accessed primary care mental health services (*P*<.001).

### Objective 3 (NEON Trial Current vs Previous User of Specialist Services)

NEON Trial participants with lifetime experience of specialist service use (N=609) were partitioned into *current specialist service use* (263/609, 43.2%) and *prior specialist service use* (346/609, 56.8%). Differences between these subgroups are investigated in Multimedia Appendix 3. After Bonferroni correction to a significance threshold of Cronbach α=.0031, the only significant difference between current and prior specialist service users was in the main mental health problem in the last month, for which current service users were more likely to be still experiencing psychosis (current: 91/263, 34.6%, vs prior: 56/346, 16.2%) and less likely to have a mood disorder (current: 81/263, 30.8%, vs prior: 134/346, 38.7%; *P*<.001).

## Discussion

### Principal Findings

This paper has presented an analysis of baseline participant characteristics in the NEON Trial and NEON-O Trial and explored their relationship with self-reported lifetime specialist mental health service use. Having used specialist services versus not having used specialist services were explained by a broad range of sociodemographic and clinical characteristics in both trials. Where there were statistically significant differences in clinical outcome data collected at baseline, specialist service users as a group always had poorer outcomes, although NEON Trial specialist service users self-identified as being further through the recovery process than people who had not used specialist services. For the NEON Trial, prior specialist service users were less likely than current specialist service users to be still experiencing psychosis as their main mental health problem and more likely to identify a mood disorder as their main mental health problem. Participants in the NEON trials were recruited from all regions of England, including in the North East and Yorkshire where there were no hospital-based recruitment sites and, hence, where participants were likely recruited through online mechanisms. The recruited participants covered all available educational levels and employment types. This provides collective evidence that the recruitment method chosen (hybrid online and offline) reached a broad range of trial participants and, hence, is advisable for trials seeking to reach underrepresented groups.

### Limitations

Both trials recruited a convenience sample with substantially more specialist service users than participants who had not used specialist services. All sociodemographic characteristics were self-rated; characteristics that could be described in state records (such as mental health service use and educational attainment) were not verified against these records. More limited information on service use was collected for the NEON-O Trial than for the NEON Trial. Participants not identifying as male or female were excluded for hypothesis testing owing to the small numbers. Although grouping ethnicity responses into 2 categories (White British and Other ethnicity) was necessary because of small numbers in most ethnicity categories other than White British, we acknowledge that this could be perceived as a reductive approach to working with ethnicity data. Some missing EQ-5D data were caused by a mismatch on the constructs of sex and gender between the requirements of an external library and the design of a demographic item. Although statistically significant relationships have been found between service use history and baseline participant characteristics, the absence of longitudinal data means that no conclusions can be drawn on causality. Although information on some protected characteristics has been collected through the demographics form, the inclusion of a broader range of demographic items drawn from the UK Equality Act [58] would have enabled an assessment of the reach of recruitment work against national legislation.

### Comparison With Prior Work

Most recruitment work for the NEON trials did not privilege service users, and we used a broad range of recruitment approaches, including paid promotion on social media platforms, intended to reach out to nonservice users. However, both trials still recruited a sample in which substantially more people had used specialist services than those who had not, despite substantial evidence that nonservice users are the majority population [24]. This is in keeping with existing research demonstrating that engaging nonservice users is more difficult than engaging service users [59]. This demonstrates that continued work is needed to understand how to engage nonservice users in mental health studies. A survey of the use of Facebook to recruit participants for health research studies found a range of studies in which the recruited sample either matched or did not match estimated population means [60]. In the NEON trials, the sample distribution for some demographic items (eg, gender) clearly did not match likely population means. Collectively, this suggests that additional controls may be needed in online recruitment work in situations where a more representative sample is required, such as the incorporation of demographic items into eligibility evaluation interfaces.

### Implications

#### Accounting for Service Use History in Change Modeling Work

The analyses presented in this paper have identified statistically significant differences between people who have used specialist mental health services and those who have not, and we might anticipate that some of these differences could influence how a DHI creates change. Medical Research Council guidance on complex intervention development recommends understanding how interventions create change, including identifying mechanisms and contextual factors [61]. Research that seeks to model change should consider the role of service use experiences in influencing how and why change happens, explicitly taking into account the experiences of people who have used mental health services and those who have not. This has the potential to enable intervention development work that accounts for service use history, such as including information tailored to service user and nonservice user perspectives. As an element of this work, interviews conducted as part of trial process evaluation work should ask about interactions between service use history and the change created by an intervention, and the findings should be reported.

#### Stratification in Trials Recruiting Service Users and Nonservice Users

A review of stratified randomization has recommended its use in small trials (<200 participants per arm) where the treatment outcome may be affected by clinical factors with a large effect on prognosis, in large trials for which interim analyses are planned with small numbers of participants, and in equivalence trials [62]. As baseline patient characteristics can predict outcomes in mental health [63] and as our findings indicate that self-reported service use history was associated with a range of baseline characteristics in both trials, we recommend that investigators consider stratified randomization on service use history for trials in which both service users and nonservice users are recruited, particularly for smaller trials as per the recommendations of the aforementioned review [62]. An advantage of this approach is that self-reported service history can be assessed through a small number of questions with objective answers, potentially limiting data collection burden for participants. Future work should examine the optimum data collection method for self-reported service use data intended for use in randomized stratification. It should consider whether stratified randomization on service use history has the potential to minimize the number of stratification variables that are required.

#### Power Calculations in Trials Recruiting Service Users and Nonservice Users

Differences between service users and nonservice users, such as poorer baseline outcomes for service users, might be associated with differences in attrition rates. Although variation in participant characteristics has not been a focus of this paper, it is notable from Tables 2 and 4 that, for all outcomes collected, sample deviations in specialist service users were equal to, or higher than, those in participants who had not used specialist services. To enable effective decision-making around power calculations, future research is needed to examine differences (if any) in attrition rates between service users and nonservice users in trials recruiting openly and to consider whether there is a marked difference in the variation of characteristics between service user and nonservice user populations on important sociodemographic and clinical characteristics. If present, sample size calculations for studies recruiting openly may need to account for this (eg, by setting minimum quotas for service users and nonservice users and by assuming differential dropout rates for these 2 subgroups). If there are differences in how change occurs for these 2 subgroups, then minimum quotas should be considered essential to support external validity.

#### Implications for Future Research With Nonservice Users

Whether service users and nonservice users have the same or different mental health experiences is an open question [34]. Our findings could be seen as consistent with either possibility because differences in participant characteristics at baseline could be explained by the impact of mental health service use (eg, specialist service treatment making mental health problems worse) or by fundamental differences in mental health experience (eg, people with more severe difficulties being more likely to use mental health services). Future studies might monitor the impact of in-study changes in service use (eg, a participant who is a nonservice user at baseline commencing an engagement with mental health services during a study) to provide evidence to disambiguate these possibilities. For the NEON trials, some in-study changes in specialist service use will be identifiable through data on statutory service use collected through 1-year retrospective Client Services Receipt Inventory data collected at the primary end point (eg, if a participant identifying as a nonservice user at baseline subsequently reports an overnight stay on a mental health ward on the 1-year Client Services Receipt Inventory form collected at the primary end point) [32].

### Conclusions

This paper has presented an analysis of baseline participant characteristics in 2 pragmatic trials of a DHI that have openly recruited people who had used specialist mental health services and those who had not. Having used specialist mental health services and not having used specialist mental health services were explained by a broad range of sociodemographic and clinical characteristics in both trials. DHIs have the potential to extend mental health service provision to people not currently using statutory mental health services, and we have argued that differences in mental health service use history have the potential to influence how mental health interventions create change in participants. Hence, for interventions targeted at populations with mixed service use histories, investigators should consider how service use should be accounted for in work to develop and evaluate new interventions.

## Appendix

Multimedia Appendix 1 Involvement plan.

Multimedia Appendix 2 Description of online forms used to collect baseline data.

Multimedia Appendix 3 Tables comparing characteristics of current and prior specialist service users in the Narrative Experiences Online Trial.

## Abbreviations

CORE-10: Clinical Outcomes in Routine Evaluation-10
CORE-OM: Clinical Outcomes in Routine Evaluation–Outcome Measure
DHI: digital health intervention
HHI: Herth Hope Index
MANSA: Manchester Short Assessment
MHCS: Mental Health Confidence Scale
NEON: Narrative Experiences Online
NEON-C: Narrative Experiences Online for people with experience of caring for people with mental health problems
NEON-O: Narrative Experiences Online for other (eg, nonpsychosis) mental health problems
NHS: National Health Service
RCT: randomized controlled trial
SMI: severe mental illness
