# Supplementary material for: Differences Between Online Trial Participants Who Have Used Statutory Mental Health Services and Those Who Have Not: Analysis of Baseline Data From 2 Pragmatic Trials of a Digital Health Intervention
Source: J Med Internet Res. 2023 Jun 27;25:e44687. doi: 10.2196/44687 (PMC10337398; doi:10.2196/44687)
Supplement: Multimedia Appendix 1 [file jmir_v25i1e44687_app1.docx]

Appendix 1 - Involvement plan

This plan was co-produced at a meeting between the lead author, and two authors not employed as researchers, and who bring substantial experience of research involvement and personal experience of mental health problems (the *lay authors*). The intention of the involvement plan is to enable meaningful involvement of the lay authors. The agreed tasks for the lay authors are as follows:

- to review the Abstract once complete, to ensure that it is as accessible as possible to people who are not professional researchers. This is because the abstract is the a likely route into the paper for people who are not professional researchers.
- to meet with the lead author to consider what Results mean once a draft is available, so that this thinking can be integrated into the Discussion. This might include generating ideas on what future research might be conducted due to the results.
- to review paper once complete, looking in particular at comprehensibility to people who are not professional researchers.
- to write and publish blog once the paper has been published. The intention of the blog is to serve as a plain English statement for the paper, so as to provide a route for the paper to be discovered by non-researchers. The blog should provide a brief summary of why it is important. Alternatively, the blog might be written by a person with lived experience of mental health problems who is not an author of the paper.

.
