# Supplementary material for: Differences Between Online Trial Participants Who Have Used Statutory Mental Health Services and Those Who Have Not: Analysis of Baseline Data From 2 Pragmatic Trials of a Digital Health Intervention
Source: J Med Internet Res. 2023 Jun 27;25:e44687. doi: 10.2196/44687 (PMC10337398; doi:10.2196/44687)
Supplement: Multimedia Appendix 2 [file jmir_v25i1e44687_app2.docx]

Appendix 2 – description of online forms used to collect baseline data

The following summarise essential information about forms used to collect demographic items and measures for participants in the NEON Trial and NEON-O Trial. Demographic items are collected once, at baseline, through a single form. The exception is two demographic items which are required to specialise questions 2 and 8 in the Manchester Short Assessment of Quality of Life (MANSA), which are collected through the same form used to collect MANSA. Where details of a form need to be specialised to a specific trial, then specialisation options are included in the text

With each form, we have summarised:

- A title and subtitle that will be visible to participants to explain its purpose
- Any necessary instructions on how to fill out the form
- The items to be included in the body of each form
- Details of legitimate values that can be entered into the form, and what needs to be completed before the form can be submitted.
- (For measures where a score is calculated) The algorithm for calculating the score, and the range of the score.

# Demographics form

**Title**: Form 1/10 – information about you

**Subtitle**: This information will help us understand who is taking part in our trials

[The response to each question should default to “Select an answer from the list”. The form should only allow submission once this has been changed to a legitimate response option for all questions].

**Items**

1. What is your age in years? [Response options: integer from 18 upwards, no upper limit]
2. What best describes your gender? [Response options: Female, Male, Other]
3. What best describes your ethnicity?

Response options: Items in italic can be selected, other items provide structure. Only one item to be selected from the following.

White
 *British*
 *Irish*
 *Gypsy or Irish Traveller*
 *Any other White background*
Mixed / Multiple ethnic groups
 *White and Black Caribbean
 White and Black African
 White and Asian
 Any other Mixed / Multiple ethnic background*
Asian / Asian British
 *Indian
 Pakistani
 Bangladeshi
 Chinese
 Any other Asian background*Black / African / Caribbean / Black British
 *African
 Caribbean
 Any other Black / African / Caribbean background*Other ethnic group
 *Arab
 Any other ethnic group*

1. What region of England do you currently live in?
   [Response options (one only): East of England, London, Midlands, North East and Yorkshire, North West, South East, South West]
2. What best describes your highest qualification? [Response options (one only): No qualifications, O-levels/GCSE or equivalent, A-levels/AS-levels/NVQ or equivalent, Degree-level qualification, Higher degree-level qualification]
3. Have you ever (including currently) used primary care mental health services, e.g. had support or medication prescribed by your GP for mental health issues, seen a GP practice counsellor, or used the Increasing Access to Psychological Therapies (IAPT) programme?
   [Response options (one only): Yes, No.]
4. Have you ever (including currently) used specialist mental healthcare services, e.g. a community mental health team, mental health in-patient ward?
   [Response options (one only): Yes, No.]
5. [NEON Trial only] Which of the following best describes the current contact you have with the NHS about your experiences of psychosis
   [Response options (one only): No contact with any NHS service, Contact with my GP, Contact with primary care counsellor, Contact with Improving Access to Psychological Therapies (IAPT), Contact with a specialist community mental health team, Currently a mental health in-patient in hospital]
6. Which of the following best describe the main mental health problem you have experienced in the last month? .
   [Response options (one only): I don’t want to say, I did not experience mental health problems, Developmental disorder such as learning disability, Eating disorder such as anorexia or bulimia, Mood disorder such as depression, anxiety or bipolar, Personality disorder such as borderline personality disorder, Schizophrenia or other psychosis such as schizo-affective disorder or delusional disorder, Stress-related disorders such as PTSD or OCD, Substance-related disorder such as alcohol or drug mis-use]

If any options other than “I did not experience mental health problems” or “I don’t want to say” are selected for question 9

1. Which of the following best describe how you are thinking about your recovery from mental health problems? These terms have been taken from a research model.
   [Response options (one only): I don’t want to say, Not yet thinking about recovery, Working on recovery, Living beyond disability]

# Primary outcome measure: Manchester Short Assessment of Quality of Life

**Title**: Form 2/10 – your quality of life

**Subtitle**: This information will help us understand your current quality of life, so that we can see how this changes during the trial.

**Items**

Pre-question 1: What is your occupation? [Response options (one only): Employed, Sheltered employment, Training or education, Unemployed, Retired]

Pre-question 2: Do you live alone or with others? [Response options (one only): Alone, With others]

1. How satisfied are you with your life as a whole today?

2. [Specialise using pre-question 1]

How satisfied are you with your [job, sheltered employment, training/education] as your main occupation?

Or if unemployed or retired…How satisfied are you with being [unemployed, retired]?

3. How satisfied are you with your financial situation?

4. How satisfied are you with the number and quality of your friendships?

5. How satisfied are you with your leisure activities?

6. How satisfied are you with your accommodation?

7. How satisfied are you with your personal safety?

8. [Specialise using pre-question 2]

How satisfied are you with the people that you live with?

Or if you live alone… How satisfied are you with living alone?

9. How satisfied are you with your sex life?

10. How satisfied are you with your relationship with your family?

11. How satisfied are you with your physical health?

12. How satisfied are you with your mental health?

**Response options**: Questions 1-12 are answered using the following satisfaction scale:

Couldn’t be worse, Displeased, Mostly dissatisfied, Mixed, Mostly satisfied, Pleased, Couldn’t be better.

For each question, the form defaults to no value having been entered, and the form can only be submitted once one of these responses has been provided against each question.

**Score:** Items in the satisfaction scale are scored sequentially 1 (Couldn’t be worse) to 7. Calculate mean of all answered questions.

**Range**: 1-7*.*

# Secondary outcome measure: CORE-10

**Title**: Form 3/10 – your recent experiences of distress

**Subtitle**: This information will help us understand your recent experiences of distressing symptoms, so that we can see how these change during the trial.

**Instructions**: This form has 10 statements about how you have been OVER THE LAST WEEK. Please read each statement and think how often you felt that way last week.

**Items**

Over the last week:

1. I have felt tense, anxious or nervous

2. I have felt I have someone to turn to for support when needed

3. I have felt able to cope when things go wrong

4. Talking to people has felt too much for me

5. I have felt panic or terror

6. I made plans to end my life

7. I have had difficulty getting to sleep or staying asleep

8. I have felt despairing or hopeless

9. I have felt unhappy

10. Unwanted images or memories have been distressing me

**Response options**: Questions 1 to 10 are answered using the following items.

*Not at all. Only occasionally. Sometimes. Often. Most or all the time*.

For each question, the form defaults to no value having been entered, and the form can only be submitted once one of these responses has been provided against each question.

**Score**: Items are scored 0 (Not at all) to 4 for all items other than 2 and 3, which are reversed. Report sum of all item scores.

**Range**: 0-40.

# Secondary outcome measure: Herth Hope Index

**Title**: Form 4/10 – your current hopefulness.

**Subtitle**: This information will help us understand how hopeful you feel at present, so that we can see how this changes during the trial.

**Instructions**: Listed below are a number of statements regarding hope. Read each statement and decide whether it applies to you personally. There are no right or wrong answers. Select the appropriate answers to indicate how often the statement has applied to you in the past week or two.

**Items**

1. I have a positive outlook toward life.

2. I have short and/or long range goals.

3. I feel all alone.

4. I can see possibilities in the midst of difficulties.

5. I have a faith that gives me comfort.

6. I feel scared about my future.

7. I can recall happy/joyful times.

8. I have deep inner strength.

9. I am able to give and receive caring/love.

10. I have a sense of direction.

11. I believe that each day has potential.

12. I feel my life has value and worth.

**Response options**: Questions 1 to 12 are answered using the following items.

Strongly disagree. Disagree. Agree. Strongly agree.

For each question, the form defaults to no value having been entered, and the form can only be submitted once one of these responses has been provided against each question.

**Score**: Items are scored 1 (Strongly disagree) to 4, other than 3 and 6 which are reversed. Calculate sum of all item scores.

**Range***:* 12-48.

# Secondary outcome measure: Mental Health Confidence Scale

**Title:** Form 5/10 – your confidence and empowerment

**Subtitle:** This information will help us understand how confident you are about your ability to deal with life challenges, so that we can see how this changes during the trial.

**Instructions**: For each item, indicate how confident you are that you could do something to help yourself right now. Rate the degree of your confidence by choosing a number from 1 to 6 where 1=very non-confident and 6=very confident.

1. Be happy

2. Feel hopeful about the future

3. Set goals for yourself

4. Get support when you need it

5. Boost your self-esteem

6. Make friends

7. Stay out of hospital

8. Face a bad day

9. Deal with losing someone to you

10. Deal with feeling depressed

11. Deal with feeling lonely

12. Deal with nervous feelings

13. Deal with symptoms related to your mental health diagnosis

14. Say no to a person abusing you

15. Use your right to accept or reject mental health treatment

16. Advocate for your needs

**Response options**: Questions 1 to 16 are answered using the numbers 1-6, where number 1 is labelled with “Very non-confident” and number 6 is labelled with “Very confident”.

For each question, the form defaults to no value having been entered, and the form can only be submitted once one of these responses has been provided against each question.

**Score**: Calculate sum of all item scores.

**Range**: 16-96.

# Secondary outcome measure: Meaning in Life Scale

**Title:** Form 6/10 – the meaning you find in your life

**Subtitle:** This information will help us to understand how much you are searching for meaning in your life, and how much you have found it, and to see how these change during the trial.

**Instructions**: Please take a moment to think about what makes your life feel important to you. Please respond to the following statements as truthfully and accurately as you can, and also please remember that these are very subjective questions and that there are no right or wrong answers.

**Items**

1. I understand my life’s meaning.

2. I am looking for something that makes my life feel meaningful.

3. I am always looking to find my life’s purpose.

4. My life has a clear sense of purpose.

5. I have a good sense of what makes my life meaningful.

6. I have discovered a satisfying life purpose.

7. I am always searching for something that makes my life feel significant.

8. I am seeking a purpose or mission for my life.

9. My life has no clear purpose.

10. I am searching for meaning in my life

**Response options**: Questions 1 to 10 are answered using the following items.

Absolutely Untrue, Mostly Untrue, Somewhat Untrue, Can’t Say True or False, Somewhat True, Mostly True, Absolutely True

For each question, the form defaults to no value having been entered, and the form can only be submitted once one of these responses has been provided against each question.

**Score**: Items are scored 1 (Absolutely Untrue) to 7, other than item 9 which is scored reversed. Calculate **presence** as mean of items 1,4,5,6 and 9*.* Calculate **search** as mean of items 2,3,7,8 and 10.

**Range**: 1-7 for presence sub-scale and 1-7 for search sub-scale.

# Health economics outcome measure: EQ5D-5L

**Title:** Form 7/10 – your health status

**Subtitle**: The following information will help us to calculate how cost-effective NEON is.

[The response to each question should default to “Select an answer from the list”. The form should only allow submission once this has been changed to a legitimate response option for all questions]..

**Instructions**: Under each heading, please select which best describes your health TODAY.

**Items**

#### 1. Mobility

Response options (one only): I have no problems in walking about, I have slight problems in walking about, I have moderate problems in walking about, I have severe problems in walking about, I am unable to walk about

#### 2. Self-care

Response options (one only): I have no problems washing or dressing myself, I have slight problems washing or dressing myself, I have moderate problems washing or dressing myself, I have severe problems washing or dressing myself, I am unable to wash or dress myself

#### 3. Usual activities (e.g. work, study, housework, family or leisure activities)

Response options (one only): have no problems doing my usual activities, I have slight problems doing my usual activities, I have moderate problems doing my usual activities, I have severe problems doing my usual activities, I am unable to do my usual activities

#### 4. Pain / Discomfort

Response options (one only): I have no pain or discomfort, I have slight pain or discomfort, I have moderate pain or discomfort, I have severe pain or discomfort, I have extreme pain or discomfort

#### 5. Anxiety / Depression

Response options (one only): I am not anxious or depressed, I am slightly anxious or depressed, I am moderately anxious or depressed, I am severely anxious or depressed, I am extremely anxious or depressed

**Score**: Response options are scored 1 (e.g. “I have no problems walking about”) to 5. Calculate sum of all items

**Range**: 5-25.
