# Supplementary material for: Differences Between Online Trial Participants Who Have Used Statutory Mental Health Services and Those Who Have Not: Analysis of Baseline Data From 2 Pragmatic Trials of a Digital Health Intervention
Source: J Med Internet Res. 2023 Jun 27;25:e44687. doi: 10.2196/44687 (PMC10337398; doi:10.2196/44687)
Supplement: Multimedia Appendix 3 [file jmir_v25i1e44687_app3.docx]

Appendix 3 – tables comparing characteristics of current and prior specialist service users in the NEON Trial

**Table A1. Baseline sociodemographic and clinical characteristics for NEON Trial participants identified as current (n=263) and prior (n=346) specialist service users**Significant p-values are highlighted in italic (α=.0031). ^†^ indicates rows merged to avoid participant identifiability where cell counts are less than 5.

|  | **Current and Prior specialist service use comparison** | | |
| --- | --- | --- | --- |
|  | **Current Specialist Service use (N=263)** | **Prior Specialist Service use (N=346)** | **p-value** |
| **Gender** n (%) |  |  | .91 |
| Female | 168(63.9) | 224(64.7) |  |
| Male | 90(34.2) | 116(33.5) |  |
| Other | 5(1.9) | 6(1.7) |  |
| **Age** years |  |  | .52 |
| Mean (SD) | 36.3(12.6) | 35.7(12.2) |  |
| **Ethnicity** n (%) |  |  | .83 |
| British White | 207(78.7) | 276(79.8) |  |
| Other Ethnicity | 56(21.3) | 70(20.2) |  |
| **Region of residence** n (%) |  |  | .25 |
| East of England | 20(7.6) | 23(6.6) |  |
| London | 56(21.3) | 64(18.5) |  |
| Midlands | 41(15.6) | 51(14.7) |  |
| North East and Yorkshire | 26(9.9) | 43(12.4) |  |
| North West | 15(5.7) | 39(11.3) |  |
| South East | 53(20.2) | 66(19.1) |  |
| South West | 52(19.8) | 60(17.3) |  |
| **Highest educational qualification** n (%) |  |  | .40 |
| No qualification | 17(6.5) | 25(7.2) |  |
| O-levels/GCSE | 38(14.4) | 62(17.9) |  |
| A-levels/AS-levels/NVQ or equivalent | 90(34.2) | 132(38.2) |  |
| Degree-level qualification | 82(31.2) | 90(26) |  |
| Higher degree level qualification | 36(13.7) | 37(10.7) |  |
| **Living status** n (%) |  |  | .13 |
| Alone | 89(33.8) | 96(27.7) |  |
| With Others | 174(66.2) | 250(72.3) |  |
| **Employment status** n (%) |  |  | .16 |
| ^†^ Employed *or* Sheltered employment | 76(28.9) | 132(38.1) |  |
| Training and education | 26(9.9) | 33(9.5) |  |
| Unemployed | 151(57.4) | 171(49.4) |  |
| Retired | 10(3.8) | 10(2.9) |  |
| **Main Mental Health Problem in last month** n(%) |  |  | *<.001* |
| Don’t want to say | 7(2.7) | 6(1.7) |  |
| Did not experience mental health problems | 7(2.7) | 11(3.2) |  |
| ^†^ Developmental disorder *or* Substance-related disorder | 6(2.2) | 16(4.6) |  |
| Eating disorder | 5(1.9) | 7(2) |  |
| Mood disorder | 81(30.8) | 134(38.7) |  |
| Personality disorder | 46(17.5) | 77(22.3) |  |
| Schizophrenia or other psychosis | 91(34.6) | 56(16.2) |  |
| Stress-related disorders | 20(7.6) | 39(11.3) |  |
| **Lifetime user of primary care mental health services** n (%) |  |  | .75 |
| **Yes** | 256 (97.3) | 339 (98) |  |
| **No** | 7 (2.7) | 7 (2) |  |
| **Current use of mental health services for psychosis** n (%) |  |  | NA |
| No contact with any NHS service | 0(0) | 74(21.4) |  |
| General Practitioner | 0(0) | 186(53.8) |  |
| Primary care counsellor | 0(0) | 42(12.1) |  |
| Improving Access to Psychological Therapies (IAPT) | 0(0) | 39(11.3) |  |
| Specialist community mental health team | 246(93.5) | 0(0) |  |
| Mental health in-patient in hospital | 17(6.5) | 0(0) |  |
| **How would you best describe your recovery?** n (%) |  |  | .23 |
| I don’t want to say | 16(6.1) | 20(5.8) |  |
| Not yet thinking about recovery | 21(8) | 42(12.1) |  |
| Working on recovery | 195(74.1) | 233(67.3) |  |
| Living beyond disability | 31(11.8) | 51(14.7) |  |

**Table A2. Baseline measures for NEON Trial participants identified as current (n=263) and prior (n=346) specialist service users**Significant p-values are highlighted in italic (α=.0031).

| **MANSA** Mean (SD) | 3.6(0.9) | 3.6(1) | .88 |
| --- | --- | --- | --- |
| **CORE-10** Mean (SD) | 22.6(7.5) | 22.9(7.7) | .66 |
| **Herth Hope Index** Mean (SD) | 28.7(6.9) | 28.7(6.8) | .93 |
| **Mental health confidence scale** Mean (SD) | 49.5(14.4) | 49.8(14.6) | .81 |
| **Meaning in Life, Presence Subscale** Mean (SD) | 3.4(1.5) | 3.4(1.4) | .84 |
| **Meaning in Life, Search Subscale** Mean (SD) | 4.7(1.4) | 4.7(1.4) | .88 |
| **EQ-5D-3L** Median (IQR) | 0.5(0.5) | 0.5(0.4) | .57 |
